# The influence of serotonin transporter polymorphisms on cortical activity: A resting EEG study

**DOI:** 10.1186/1471-2202-12-33

**Published:** 2011-04-20

**Authors:** Tien-Wen Lee, Younger WY Yu, Chen-Jee Hong, Shih-Jen Tsai, Hung-Chi Wu, Tai-Jui Chen

**Affiliations:** 1 Department of Psychiatry, Chang Gung Memorial Hospital, Taoyuan County, Taiwan; 2 College of Medicine, Chang Gung University, Taoyuan County, Taiwan; 3 Yu's Psychiatric Clinic, Kaohsiung, Taiwan; 4 Department of Psychiatry, Taipei Veterans General Hospital, Taipei, Taiwan; 5 School of Medicine, National Yang-Ming University, Taipei, Taiwan; 6 Kai-Suan Psychiatric Hospital, Kaohsiung, Taiwan; 7 Department of Psychiatry, E-DA Hospital, Kaohsiung County, Taiwan; 8 Department of Occupational Therapy, I-Shou University, Kaohsiung County, Taiwan

**Keywords:** electroencephalography (EEG), power spectrum, *5-HTTLPR*, *5-HTTVNTR*, polymorphism

## Abstract

**Background:**

The serotonin transporter gene (*5-HTT*) is a key regulator of serotonergic neurotransmission and has been linked to various psychiatric disorders. Among the genetic variants, polymorphisms in the *5-HTT *gene-linked polymorphic region (*5-HTTLPR*) and variable-number-of-tandem-repeat in the second intron (*5-HTTVNTR*) have functional consequences. However, their genetic impact on cortical oscillation remains unclear. This study examined the modulatory effects of *5-HTTLPR *(L-allele carriers vs. non-carriers) and *5-HTTVNTR *(10-repeat allele carriers vs. non-carriers) polymorphism on regional neural activity in a young female population.

**Methods:**

Blood samples and resting state eyes-closed electroencephalography (EEG) signals were collected from 195 healthy women and stratified into 2 sets of comparisons of 2 groups each: L-allele carriers (*N *= 91) vs. non-carriers for *5-HTTLPR *and 10-repeat allele carriers (*N *= 25) vs. non-carriers for *5-HTTVNTR*. The mean power of 18 electrodes across theta, alpha, beta, gamma, gamma1, and gamma2 frequencies was analyzed. Between-group statistics were performed by an independent t-test, and global trends of regional power were quantified by non-parametric analyses.

**Results:**

Among *5-HTTVNTR *genotypes, 10-repeat allele carriers showed significantly low regional power at gamma frequencies across the brain. We noticed a consistent global trend that carriers with low transcription efficiency of 5-HTT possessed low regional powers, regardless of frequency bands. The non-parametric analyses confirmed this observation, with *P *values of 3.071 × 10^-8 ^and 1.459 × 10^-12 ^for *5-HTTLPR *and *5-HTTVNTR*, respectively.

**Conclusions and Limitations:**

Our analyses showed that genotypes with low 5-HTT activity are associated with less local neural synchronization during relaxation. The implication with respect to genetic vulnerability of 5-HTT across a broad range of psychiatric disorders is discussed. Given the low frequency of 10-repeat allele of *5-HTTVNTR *in our research sample, the possibility of false positive findings should also be considered.

## Background

Serotonin or 5-hydroxytryptamine (5-HT) is a monoamine derived from tryptophan and is abundant in the gastrointestinal tract, platelets, and the central nervous system. Serotonin acts as a neurotransmitter in the brain. 5-HT transporter (5-HTT) plays a central role in regulating 5-HT synaptic function by transporting the neurotransmitter serotonin from synaptic spaces into pre-synaptic neurons. Among the genetic variants of 5-HTT, polymorphisms in the 5-HTT gene-linked polymorphic region (*5-HTTLPR*) and variable-number-of-tandem-repeat in the second intron (*5-HTTVNTR*) have functional consequences and are of particular interest. Although it is still debatable [1], the functional polymorphisms of the *5-HTT *gene have been reported to influence susceptibility to traumatic experience and risk for various forms of depression, anxiety, psychosis, alcoholism, and suicidal behaviors [2-12]. Further, low expression of 5-HTT is associated with increased adverse effects and reduced treatment response to antidepressants [13-15].

The differential neural responses caused by *5-HTTLPR *polymorphism (S: S-allele, L(A/G): L-allele) have been under active study. The L-/S-allele reflect a 43-bp insertion/deletion in the 5-HTT-linked polymorphic region, wherein the long variant is more than twice as active as the short variant [16]. A single nucleotide polymorphism of A/G (rs25531) within the L-allele was reported to lead to two functionally different variants, with the transcriptional activity of the less common G substitution similar to that of the S-allele [17]. Risk allele carriers (S or L(G)) with low expression of 5-HTT showed increased amygdala reactivity to masked emotional faces and to overtly sad faces [18,19]. In the resting state, the subjects carrying homozygous S-alleles showed increased and decreased cerebral blood flow in the amygdala and in the ventromedial prefrontal cortex, respectively [20]. Enhanced basal metabolism in fronto-limbic structures for carriers homozygous for S-alleles was regarded to enhance the susceptibility for developing an anxiety-depression spectrum disorder [21].

Compared with *5-HTTLPR*, the neural mechanism influenced by *5-HTTVNTR *polymorphism (e.g., 10-repeat and 12-repeat) is much less clear, with the 10-repeate allele bearing lower transcription efficiency than the 12-repeat one. Similar to the imaging studies of *5-HTTLPR*, Bertolino et al. noticed that the *5-HTTVNTR *genotype predicted amygdala activity to threatening stimuli [22]. However, the findings of *5-HTTVNTR *and *5-HTTLPR *seemed not always convergent. For example, the studies on *5-HTTLPR *and *5-HTTVNTR *have shown that *5-HTTLPR *genotypes did not differentiate the degree of harm avoidance but *5-HTTVNTR *did [23]. Preferential transmission of *5-HTTLPR *not *5-HTTVNTR *genotypes has been reported for autism [24,25]. A tendency toward an increase in the *5-HTTLPR *L-allele and VNTR 10-repeat allele, not 12-repeat, has been observed in suicide victims [26]. The inconsistency raised a concern that the consequence of *5-HTTLPR *and *5-HTTVNTR *genotypes (although both affect the transcription efficiency of 5-HTT) cannot be fully explained by differential serotonin levels at the neural cleft. Thus, whether the neuroimaging findings based on *5-HTTLPR *can be extrapolated to *5-HTTVNTR *warrants empirical examination.

The serotonin system exerts a complicated impact on the neuromatrix of electrocortical activities. Since the function of 5-HTT is related to the clearance of serotonin in the neural cleft, it is pertinent to refer to the pharmaco-EEG studies contingent on enhanced (e.g. administration of serotonin reuptake inhibitors) or reduced (e.g. acute tryptophan depletion) serotonergic neurotransmission. Acute enhancement of serotonin level produces a widespread decrease in slow and medium frequency activity and an increase in fast frequency activity on electroencephalography (EEG) scans; however, a reduced serotonin level is also associated with decreased delta/theta and increased alpha/beta powers [27,28]. Knott et al. observed that the reduction in central serotonin caused a significant and widespread increase in slow wave delta amplitude [29]. Administration of serotonin reuptake inhibitors is related to reduced slow wave and increased alpha or beta activities [30,31] but negative EEG alteration has also been reported [32]. In summary, the extant literature seems inconclusive with respect to the interaction between serotonergic neurotransmission and electrocortical oscillation. The effects of global serotonin level on event-related potentials are more consistent but showed contextual dependency. For example, acute tryptophan depletion is associated with suppressed P50/N1 enhancement to target, decreased intensity dependence of N1m/P2m in auditory cortex, reduced N2 amplitude in mismatch negativity and attenuated N1P2/P300 amplitudes in oddball design (for patients of bipolar disorders; without EEG power change) [33-36], while negative findings are noticed in somatosensory stimulation and episodic memory retrieval [37,38]. Nevertheless, it was also reported that no significant alterations in auditory evoked potentials and background EEG power/frequency followed acute tryptophan depletion [39].

Thus far, only a few studies have investigated the change in quantitative EEG (qEEG) indices stratified by *5-HTTLPR *and *5-HTTVNTR *genetic polymorphisms. The risk allele of *5-HTTLPR *is related to mildly increased right frontal asymmetry and imposes no direct effect on target N1 and novelty N1 potentials [40,41]. The more active variant of serotonin transporter is associated with enhanced mismatch negativity and decreased P300 components [42]. Whether the differential expression of the serotonin transporter alters neural characteristics in the resting state remains unexplored. This study used EEG to investigate the effect of *5-HTTLPR and 5-HTTVNTR *on local brain dynamics in the resting state. Resting EEG carries abundant information predictive of performance on several neuro-psychological tasks and even the early stage of Alzheimer's disease or the treatment response in major depressive disorders [43-48]. Our design also examined an issue of whether the neural manifestation of *5-HTTLPR *and *5-HTTVNTR *polymorphisms would converge. Since the gender difference has been noticed to moderate the association between the polymorphism of the serotonin transporter and various phenotypes [49-51], we restricted this research sample to women.

## Materials and methods

### Subjects

In this study, 208 right-handed healthy young women of a nursing college were enrolled. Their ages ranged from 19 to 21 years. Neurological and physical examinations were performed by licensed medical doctors, while their psychiatric condition was evaluated by licensed psychiatrists following a semi-structural interview process. Those with a history of substance abuse, psychiatric disease, or major medical or neurological disorders were excluded. Only those who had been medication-free, including birth control pills, for at least 2 weeks were enrolled. This project was approved by the institutional ethics committee, conforming with The Code of Ethics of the World Medical Association. Informed consent was obtained from all participants before beginning the investigation. Based on the exclusion criteria, 13 participants were not eligible and 195 participants were included in the analysis.

### EEG recordings and analyses

All participants underwent a 3-minute conventional, eyes-closed, awake, digital EEG scan after a 5-minute habituation to the experimental environment (Brain Atlas III computer, Biologic System Company, Chicago). Recordings followed the standard of the international 10-20 system with ear-linked reference at a 256 Hz sampling rate and impedance below 3 kΩ [52]. The artifact of vertical eyeball movement was detected from electrodes placed above and below the right eye, with the horizontal analog derived from electrodes placed at the left outer canthus. The frequency bands were defined as follows: theta, 4-8 Hz; alpha, 8-12 Hz; beta, 12-24 Hz; gamma, 25-60 Hz; gamma1, 25-35 Hz; and gamma2, 35-60 Hz. EEG traces with artifacts were deleted by experienced EEG researchers. Eighteen electrodes, namely, F7, F3, Fz, F4, F8, T3, C3, Cz, C4, T4, T5, P3, Pz, P4, T6, O1, Oz, and O2 were included in the analyses. We used Fast Fourier Transform to derive the mean EEG power (unit: μV^2^).

### Genotyping of 5-HTTLPR and 5-HTTVNTR polymorphisms

Genomic DNA was isolated from peripheral leukocytes and amplified using polymerase chain reaction, with the primers 5-HTTLPR-3: ATGCCAGCACCTAACCCCTAATG plus 5-HTTLPR-2: GAGGGACTGAGCTGGACAACCAC [16], and 5-HTTVNTR-F: GTCAGTATCACAGGCTGCGAG plus 5-HTTVNTR-B: TGTTCCTAGTCTTACGCCAGTG [8]. Polymorphisms of *5-HTTLPR *and *5-HTTVNTR *were identified using agarose-gel electrophoresis. The sizes of the S- and L-alleles for *5-HTTLPR *were 469-470 bp and 511-513 bp, respectively, and the sizes of the 10-repeat and 12-repeat alleles for *5-HTTVNTR *were 267 bp and 300 bp, respectively.

### Statistical analyses

Pair-wise linkage disequilibrium and Hardy-Weinberg equilibrium were analyzed using ARLEQUIN 2.000 [53]. We performed 2 independent calculations of gene-brain interaction, and the participants were categorized into 2 groups according to the genotypes of *5-HTTLPR *and *5-HTTVNTR*. For *5-HTTLPR*, the stratification was based on L-allele carriers (L/L and L/S) and non-carriers (S-allele homozygotes). For *5-HTTVNTR*, the subjects were grouped into 10-repeat allele carriers (10/10 and 10/12) and non-carriers (12-repeat allele homozygotes). An independent 2-sample t-test that assumed unequal variance was performed to elucidate the channel-frequency pairs with values of mean power showing significant between-group differences. Two-way ANOVA was carried out to investigate the interaction between the genotypes of *5-HTTLPR *and *5-HTTVNTR *on the cortico-electrical power. For each test set in this study, the criterion for significance was set at *P *< 0.05, two-tailed. We assumed the independency of each frequency band and performed a Bonferroni correction based on the formula *P *= 1 - (1 - 0.05)^1/n^, where n equals the number of comparisons. For each comparison, we reported both the P value < 0.01 and the P value adjusted for multiple comparisons (0.0028 when n = 18), in case the Bonferroni correction is too stringent since the cortical electrical activities are interactive, not totally independent.

To test whether there was a global trend difference in the mean power across regions and frequency bands between genotyped groups, we performed non-parametric analyses. Our null hypothesis assumed that the probability of a certain index (i.e., mean power) for a particular electrode at a specific frequency band, group one is greater than group two equals the probability that group two is greater than group one (i.e., the probability was 0.5). The probability to obtain j or more "group one > group two" indices by chance can be calculated using the following formula: , where s is the total number of comparisons (one comparison for each electrode-frequency couple, e.g., F3-alpha; s = 18 × 6 when taking all the electrode(18)-frequency(6) pairs into account).

Power analysis was conducted according to 2 fundamental perspectives [54]: (1) Estimate the sample number required to reach fixed type I and type II errors in the independent 2-sample t-test. (2) Estimate the z-score of power under fixed type I error and sample statistics (mean, variance and sample number). We performed power analyses for each electrode-frequency pair for *5-HTTLPR *and *5-HTTVNTR*. Interested readers may refer to the cited reference for mathematical details [54].

## Results

The *5-HTTLPR *genotypes of the participants included L/L (*N *= 16), S/L (*N = *75), and S/S (*N *= 104), and these were distributed in Hardy-Weinberg equilibrium (χ^2 ^= 0.226, *P *= 0.634). The subjects were divided into 2 groups: 104 S-allele homozygotes and 91 L-carriers (S/L and L/L). The *5-HTTVNTR *genotypes of the participants included 12/12 (*N *= 170), 12/10 (*N = *24), and 10/10 (*N *= 1) and were also distributed in Hardy-Weinberg equilibrium (χ^2 ^= 0.024, *P *= 0.878). The subjects were divided into 2 groups: 170 12-repeat allele homozygotes and 25 10-repeate allele carriers (12/10 and 10/10). The haplotype analysis detected no significant linkage disequilibrium between the 5-HTTLPR and 5-HTTVNTR polymorphisms (p = 0.237), which replicated previous observation [55].

**Figure 1:**
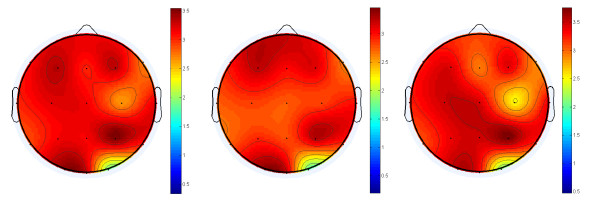
**The topography of t-statistics of mean power differences at gamma frequencies, from the comparison of the group "12-repeat allele homozygotes" minus the group "10-repeat allele carriers" for *5-HTTVNTR***. Left: gamma, middle: gamma1, right: gamma2.

As to the between-group comparisons for *5-HTTLPR *and *5-HTTVNTR*, the values and statistics of mean power over 18 channel pairs across 6 frequency bands are summarized in Tables 1 and 2, respectively. For the independent t-test based on *5-HTTLPR *genotypes (L-allele carriers vs. non-carriers), none of the electrode-frequency pairs reached statistical significance. However, for *5-HTTVNTR *genotypes (10-repeat allele carriers vs. non-carriers), the electrode-frequency pairs showing significant genotype effect aggregated at gamma frequencies and were widely distributed across the brain. Since EEG gamma power is susceptible to muscular artifacts, we plotted the topography of t-statistics for between-group comparisons for *5-HTTVNTR *(Figure 1). The strongest peaks were away from the bi-temporal region, indicating that the significant results did not likely originate from electromyographical activities of the musculus temporalis, masseter muscle, etc.

**Table 1:** Comparison of regional mean power in *5HTTLPR *L-allele carriers (upper row) and non-carriers (S allele homozygotes; lower row) for each EEG channel

	theta	alpha	beta	gamma	gamma1	gamma2
F7	12.26 (11.27)	21.56 (20.07)	3.19 (3.83)	1.12 (2.35)	1.54 (2.93)	0.95 (2.14)

	10.74 (8.29)	19.49 (18.55)	3.01 (2.26)	0.85 (1.03)	1.21 (1.19)	0.70 (0.98)

F3	19.63 (16.35)	37.84 (34.03)	4.55 (4.73)	0.93 (1.33)	1.61 (2.35)	0.66 (0.98)

	17.07 (13.07)	36.61 (38.50)	4.52 (3.66)	0.84 (0.87)	1.43 (1.23)	0.61 (0.77)

Fz	27.91 (22.14)	52.89 (46.77)	5.48 (5.43)	0.97 (1.22)	1.58 (2.28)	0.73 (0.96)

	24.65 (18.95)	51.37 (52.85)	5.66 (5.06)	0.93 (0.85)	1.40 (1.15)	0.74 (0.90)

F4	23.23 (18.72)	43.37 (37.89)	5.17 (5.02)	1.11 (1.63)	1.90 (2.74)	0.80 (1.28)

	20.10 (15.43)	43.28 (46.09)	5.35 (4.49)	1.02 (1.05)	1.72 (1.52)	0.74 (0.92)

F8	12.43 (9.98)	20.93 (17.84)	3.10 (2.84)	1.01 (1.82)	1.45 (2.38)	0.84 (1.61)

	10.63 (7.90)	21.54 (23.04)	3.03 (2.28)	0.83 (1.07)	1.18 (1.15)	0.69 (1.05)

T3	13.95 (12.03)	26.45 (28.25)	4.79 (5.09)	1.77 (2.94)	2.34 (3.73)	1.54 (2.69)

	11.61 (8.74)	24.59 (20.44)	4.26 (3.44)	1.10 (1.13)	1.55 (1.39)	0.92 (1.05)

C3	27.28 (21.80)	52.91 (51.58)	6.26 (5.59)	1.12 (1.43)	1.88 (2.30)	0.82 (1.16)

	23.47 (17.52)	49.63 (45.78)	6.47 (5.25)	0.96 (0.90)	1.66 (1.33)	0.68 (0.79)

Cz	33.54 (26.92)	63.34 (58.95)	6.33 (6.12)	0.95 (1.26)	1.70 (2.23)	0.65 (0.97)

	29.01 (21.98)	60.67 (61.58)	6.36 (5.38)	0.82 (0.71)	1.62 (1.62)	0.50 (0.42)

C4	28.69 (23.64)	57.32 (53.89)	6.53 (5.85)	1.07 (1.32)	1.86 (2.25)	0.75 (1.02)

	24.19 (18.01)	53.81 (50.71)	6.50 (5.15)	0.92 (0.88)	1.64 (1.31)	0.63 (0.75)

T4	14.91 (12.54)	27.17 (24.27)	5.42 (6.34)	1.99 (3.67)	2.58 (4.14)	1.75 (3.50)

	13.00 (9.96)	28.14 (25.30)	4.71 (3.76)	1.33 (1.88)	1.87 (2.21)	1.12 (1.76)

T5	17.00 (15.02)	48.72 (54.64)	5.20 (4.91)	1.15 (2.13)	1.67 (2.75)	0.94 (1.94)

	15.82 (19.68)	62.30 (100.16)	6.87 (14.47)	0.88 (1.08)	1.40 (1.52)	0.67 (0.93)

P3	26.87 (23.61)	68.13 (73.30)	7.05 (6.07)	0.85 (0.97)	1.53 (1.69)	0.58 (0.71)

	23.16 (19.05)	68.06 (65.39)	7.96 (9.69)	0.78 (0.69)	1.46 (1.20)	0.51 (0.51)

Pz	32.34 (26.84)	77.32 (77.78)	7.36 (6.55)	0.88 (0.98)	1.58 (1.67)	0.60 (0.76)

	27.80 (22.29)	78.06 (76.74)	7.66 (8.45)	0.76 (0.58)	1.45 (1.05)	0.49 (0.41)

P4	26.28 (21.78)	71.78 (71.84)	7.30 (5.94)	0.86 (0.93)	1.54 (1.51)	0.59 (0.73)

	23.45 (18.50)	78.70 (84.64)	8.49 (12.28)	0.76 (0.59)	1.44 (1.05)	0.49 (0.44)

T6	19.07 (16.11)	82.44 (96.34)	6.93 (6.88)	1.32 (3.07)	2.02 (4.26)	1.05 (2.62)

	17.75 (16.44)	89.91 (105.76)	8.46 (17.75)	0.92 (0.87)	1.55 (1.42)	0.67 (0.69)

O1	26.89 (24.71)	129.21 (140.92)	9.33 (8.05)	1.19 (1.61)	1.91 (2.24)	0.90 (1.40)

	23.93 (26.70)	149.02 (232.99)	13.68 (33.71)	1.18 (1.90)	2.03 (2.93)	0.84 (1.55)

Oz	26.38 (21.76)	148.51 (149.64)	10.17 (8.30)	1.16 (1.62)	1.89 (1.94)	0.86 (1.55)

	23.59 (25.14)	159.84 (239.83)	13.67 (32.34)	0.98 (0.94)	1.77 (1.82)	0.66 (0.66)

O2	32.23 (26.29)	118.62 (121.38)	12.03 (10.49)	1.94 (2.32)	3.19 (3.71)	1.44 (1.81)

	29.96 (24.62)	128.54 (148.79)	14.84 (25.75)	2.11 (3.09)	3.50 (4.85)	1.56 (2.43)

The mean power (×100) was expressed as "mean (STD)"

None of the between-group differences reached statistical significance (*P *< 0.01)

**Table 2:** Comparison of regional mean power of *5HTTVNTR *10-repeat allele carriers (lower row) and non-carriers (12-repeat allele homozygotes; upper row) for each EEG channel

	theta	alpha	beta	gamma	gamma1	gamma2
F7	12.34 (11.39)	21.47 (19.46)	3.31 (3.33)	1.03 (1.80)*	**1.45 (2.22)**	0.86 (1.65)*

	11.33 (7.85)	18.75 (14.42)	2.54 (1.34)	**0.54 (0.41)**	**0.86 (0.54)**	**0.41 (0.37)**

F3	19.60 (17.62)	38.50 (36.18)	4.81 (4.53)	0.94 (1.11)*	1.62 (1.87)*	0.67 (0.88)*

	16.86 (12.45)	31.66 (25.56)	3.54 (2.12)	**0.52 (0.37)**	**0.95 (0.61)**	**0.35 (0.30)**

Fz	28.09 (23.74)	53.91 (49.44)	5.90 (5.47)	**1.00 (1.05)**	**1.58 (1.79)**	0.76 (0.96)

	24.97 (19.24)	46.74 (37.98)	4.63 (3.10)	**0.61 (0.55)**	**0.98 (0.71)**	0.46 (0.60)

F4	23.04 (18.98)	44.94 (41.90)	5.56 (4.93)	1.13 (1.36)*	1.91 (2.24)*	0.82 (1.11)*

	20.68 (16.11)	37.52 (30.52)	4.30 (2.58)	**0.63 (0.44)**	**1.16 (0.74)**	**0.42 (0.37)**

F8	12.38 (10.52)	21.94 (20.52)	3.24 (2.75)	0.93 (1.45)	1.35 (1.83)	0.77 (1.32)

	12.01 (9.66)	19.45 (15.16)	2.63 (1.49)	0.60 (0.61)	0.93 (0.75)	0.46 (0.57)

T3	14.19 (16.15)	26.67 (24.93)	4.80 (4.68)	**1.49 (2.24)**	2.02 (2.80)	**1.28 (2.07)**

	12.80 (10.05)	25.79 (20.02)	3.76 (1.96)	**0.84 (0.81)**	1.30 (1.17)	**0.65 (0.69)**

C3	27.71 (29.55)	53.28 (48.45)	6.75 (6.07)	1.08 (1.18)*	1.85 (1.87)	0.77 (0.98)*

	25.33 (20.31)	46.20 (35.55)	5.34 (3.20)	**0.66 (0.45)**	1.29 (0.84)	**0.41 (0.33)**

Cz	33.63 (31.07)	64.00 (59.68)	6.66 (6.00)	0.94 (1.07)*	1.75 (1.95)	0.62 (0.86)*

	29.69 (22.25)	53.44 (40.54)	5.37 (3.59)	**0.57 (0.41)**	1.20 (0.92)	**0.32 (0.25)**

C4	28.74 (28.07)	57.95 (52.20)	6.95 (5.94)	1.03 (1.11)	1.85 (1.84)	0.71 (0.87)

	27.91 (24.38)	51.69 (40.37)	5.71 (3.15)	0.74 (0.66)	1.30 (0.85)	0.52 (0.77)

T4	15.19 (14.44)	28.78 (24.44)	5.32 (5.30)	1.66 (2.84)*	**2.26 (3.24)**	1.42 (2.70)*

	16.80 (16.04)	29.65 (25.92)	4.11 (2.42)	**0.79 (0.72)**	**1.29 (1.08)**	**0.59 (0.60)**

T5	18.12 (25.17)	57.31 (82.22)	6.38 (11.20)	1.06 (1.65)	1.60 (2.17)	**0.85 (1.50)**

	15.22 (15.74)	36.83 (33.93)	4.13 (2.60)	0.60 (0.64)	1.02 (0.95)	**0.43 (0.53)**

P3	28.48 (43.05)	69.57 (68.77)	7.95 (8.77)	0.85 (0.83)*	**1.57 (1.45)**	0.57 (0.62)*

	26.28 (26.99)	63.99 (52.76)	6.13 (3.80)	**0.54 (0.38)**	**1.09 (0.68)**	**0.32 (0.28)**

Pz	34.05 (46.94)	80.60 (77.37)	7.94 (8.20)	0.86 (0.79)*	1.60 (1.39)	0.57 (0.60)*

	30.61 (26.13)	70.01 (55.88)	6.25 (4.14)	**0.55 (0.41)**	1.14 (0.78)	**0.31 (0.27)**

P4	28.03 (35.61)	78.02 (79.29)	8.41 (10.35)	0.86 (0.78)*	1.59 (1.32)*	0.57 (0.60)*

	26.20 (24.85)	68.60 (54.93)	6.33 (3.94)	**0.50 (0.33)**	**1.03 (0.60)**	**0.28 (0.23)**

T6	20.40 (24.43)	88.57 (102.61)	8.08 (13.92)	**1.17 (2.18)**	**1.87 (3.09)**	**0.89 (1.85)**

	20.38 (22.51)	81.47 (78.64)	6.23 (4.30)	**0.62 (0.44)**	**1.14 (0.71)**	**0.41 (0.35)**

O1	27.77 (34.60)	141.42 (193.68)	12.16 (25.25)	1.26 (1.77)*	2.08 (2.62)*	0.92 (1.49)*

	25.00 (24.41)	97.91 (96.68)	7.41 (4.57)	**0.60 (0.43)**	**1.14 (0.63)**	**0.39 (0.38)**

Oz	26.95 (27.79)	159.87 (203.96)	12.51 (24.27)	1.10 (1.29)*	1.90 (1.87)*	0.77 (1.15)*

	26.98 (32.25)	124.16 (103.23)	8.68 (7.13)	**0.62 (0.47)**	**1.19 (0.72)**	**0.39 (0.39)**

O2	34.21 (39.13)	126.89 (137.19)	13.81 (20.07)	2.13 (3.45)	3.34 (4.18)	1.65 (3.59)

	33.42 (26.24)	101.88 (81.39)	12.03 (9.90)	2.22 (2.88)	3.61 (4.53)	1.66 (2.33)

The mean power (×100) was expressed as "mean (STD)"

The statistical challenge between *5HTTVNTR *10-repeat carriers and non-carriers with *P *< 0.01 is marked in bold (two-sided)

The threshold of *P *value after Bonferroni correction is 0.0028; *P < 0.0028

We performed non-parametric analyses to examine the genetic effect on the global trend. Since the independent samples t-test demonstrated significance at gamma frequency bands for *5-HTTVNTR*, only the regional power at frequencies theta, alpha, and beta were included in the analysis of *5-HTTVNTR*. In contrast, all electrode-frequency pairs were incorporated in the analysis of *5-HTTLPR*. We observed that 82 of 108 (18 electrodes × 6 frequency bands) and 51 of 54 electrode-frequency pairs for *5-HTTLPR *and *5-HTTVNTR *showed the same signs after between-group subtraction. Our non-parametric analyses confirmed a global trend across the brain: S-allele homozygotes of *5-HTTLPR *and 10-repeat allele carriers of *5-HTTVNTR *possess lower regional power, with respective *P *values 3.071*10^-8 ^and 1.459*10^-12^. Our two-way ANOVA analyses showed no signification interaction (*P *< 0.01). In addition, since only 9 subjects carried the allelic combination of S-allele of *5-HTTLPR *and 10-repeat of *5-HTTNVTR*, we did not conduct further statistical comparisons based on haplotypes, which could be skewed by the imbalanced sample distribution.

Our power analyses showed that for *5-HTTLPR*, we needed 511, 665, 766 and 857 subjects to have 2.5, 5.0, 7.5 and 10.0 percent electrode-frequency pairs to achieve type 1 error 0.01 and power 0.80, respectively; whereas for *5-HTTVNTR *counterparts, 197, 219, 242 and 261 subjects were required (perspective I). Given the channel statistics and type 1 error 0.01, the highest power was 0.66 based on the categorization according to *5-HTTLPR *genotypes, while 33 electrode-frequency pairs possessed power value greater than 0.80 based on the categorization according to *5-HTTVNTR *genotypes (perspective II). The power analyses were concordant with our observation in the 2-sample t-tests, and endorsed the application of non-parametric analysis, such as demonstrated in this study, to unveil the data structure difficult to be quantified by statistical methods derived from normal distributions.

## Discussion

The serotonin transporter (5-HTT) regulates 5-HT synaptic neurotransmission, which in turn exerts diverse influences on an organism from embryogenesis through adulthood [56-62]. A wide variety of psychiatric conditions are associated with the functional polymorphisms of the *5-HTT *gene, such as anxiety, depression, psychosis, alcoholism, obsessive-compulsive disorder, and suicidality [2-12,63]. This study investigated 2 common functional polymorphisms of 5-HTT, namely, *5-HTTLPR *and *5-HTTVNTR*, in order to understand their genetic contribution to resting brain dynamics. Less efficient transcription and consequently low 5-HTT availability were associated with the S-allele (S) of *5-HTTLPR *and with the 10-repeat allele of *5-HTTVNTR *when compared to their polymorphic counterparts of the L-allele (L) and 12-repeat allele. We discovered a consistent global trend for *5-HTTLPR *and *5-HTTVNTR*: the allele with low transcription efficiency was associated with low spectral power. For *5-HTTVNTR*, the statistical comparison reached significance at gamma frequencies for "10-repeat allele vs. non-carriers" across the brain.

*5-HTT *polymorphism affects synaptic serotonin availability and modulates event-related potentials. For example, carriers of more active gene variants are reported to have increased brain potential in mismatch negativity and decreased potential in P300 [42,64]. The extant literature regarding serotonin level, EEG neuromatrix, and behavioral phenotypes, nevertheless, seems to not converge well, with some reports remaining hard to reconcile. Individuals homozygous for the L-allele of *5-HTTLPR *exhibit a stronger intensity dependence on auditory-evoked potentials [65], implying a low serotonin level in the brain which agrees well with the innately high transporter activity [16,66,67]. Studies on serotonin metabolites in the cerebrospinal fluid have shown that people with low serotonin level have a substantially high risk for suicide [68]. However, genetic association studies have revealed that it is the carriers with the S-allele, not those with the L-allele, of *5-HTTLPR *who possess a higher suicide risk [69]. Various maneuvers, i.e., applying a transporter inhibitor or tryptophan depletion, have been applied to adjust the serotonin level in the central nervous system but the consequent alterations in EEG dynamics have been divergent, with both inconsistent and negative results [27,28,30-32,36]. Thus far, only 1 study has addressed the impact of *5-HTTLPR *polymorphism on quantitative EEG (qEEG); that study focused on frontal electrical asymmetry [40]. The reason that our observed global trend in spectral power change was not observed in previous research may originate from fundamental differences in the approach and design. Several accounts are discussed below.

Lesch et al. showed that carriers of the S-allele of *5-HTTLPR *have higher anxiety-related traits than homozygotes of the L-allele in healthy subjects [12]. Is it possible that our finding of reduced spectral power in individuals with the S-allele was mediated by a different anxiety trait? Knyazev et al. analyzed the participants of a different anxiety disposition and observed increased spectral power of all EEG bands in subjects with high-anxiety traits when they faced an uncertainty condition [70]. It was unfortunate that Knyazev et al.'s results were inconsistent with our findings. Is it possible that our participants of S-allele homozygotes for *5-HTTLPR *or 10-repeat allele carriers for *5-HTTVNTR *were in a higher anxiety state during the EEG recording? The qEEG studies investigating anxiety state showed enhanced power of high frequency components [71], which was replicated in the condition of induced worry for patients with generalized anxiety disorders [72]. Again, the directionality was contrary to our observation. Different arousal level is another possibility; however, heightened arousal was noticed to reduce alpha power and to increase gamma power [73,74]. We, therefore, regard our finding as novel and propose a simple and unified mechanism to account for our results: genetic polymorphism contributing to lower 5-HTT activity is associated with less synchronization of regional neural organization and consequently with smaller spectral power, regardless of frequency bands. This interpretation is simply a straightforward inference from the observed results.

It is acknowledged that ascending serotonergic projections from the raphe nuclei to wide brain areas influence cortical functioning. Most literature is focused on the concentration of 5-HT, bioavailability of various 5-HT receptors, 5-HT degradation, and the complicated interplay between intracellular cascades and the accompanying up-/down-regulation. It is often ignored that serotonin is a neurotrophic factor affecting neural architecture and also a morphogen commencing its influence during early embryogenesis. 5-HT induces neurogenesis and neuronal differentiation, affects neuronal migration, and inhibits the mobility of the growth cone [56-59]. Structural imaging studies have highlighted that the *5-HTTLPR *S-allele is associated with reduced volume at the hippocampus, caudate nucleus, and prefrontal cortex, and deceased white matter integrity over the fronto-limbic pathway [75-77]. In adulthood, the serotonergic system plays a neurotrophic role in the dentate gyrus, which might be relevant to depression recovery [60-62]. From embryo to adult, serotonergic neurotransmission exerts diverse influences on the neural architecture and associated brain function. We speculated that the *5-HTTLPR *S-allele and *5-HTTVNTR *10-repeat allele contributed to less synchronized neural circuits at the local cortical region. The detrimental effects on neural architecture at both the local and inter-regional levels might explain why the S-allele of *5-HTTLPR *manifests as a general vulnerable factor in so many psychiatric conditions [2-12,63]. *5-HTT *polymorphism is reported to deviate electrocortical activity relevant to attention and automatic perceptual detection [41,42]. Since gamma activity has been suggested as correlates of attention and sensory binding [78-81], the significant differences in gamma frequency bands for *5-HTTVNTR *polymorphism warrant further study to replicate and extend.

Although both *5-HTTLPR *and *5-HTTVNTR *are functional polymorphisms of the serotonin transporter, studies that incorporated both genotypes did not always unveil a consistent association with human phenotypes. For example, preferential transmission of *5-HTTLPR *but not *5-HTTVNTR *genotypes was reported for autism [24,25]. A tendency toward an increase in the *5-HTTLPR *allele L and VNTR allele 10-repeat, not 12-repeat, was observed in suicide victims [26]. The degree of harm avoidance was differentiated by *5-HTTLPR *genotypes but not by *5-HTTVNTR *[23]. Our result provided empirical evidence that the impact of *5-HTTLPR *and *5-HTTVNTR *was quite consistent at the neural level (endo-phenotype). We suggest that future studies recruit both genotypes as an internal validation process, particularly when the statistics are in the weak to moderate range. We also suggest that studies involving an activation task attempt the strategy of subtracting baseline qEEG indices, which might boost the sensitivity of between-genotype comparisons. Since sex and age have been observed to effect many EEG indices and, gender difference has been noticed to moderate the association between the 5-HTT polymorphism and various phenotypes [49-51,82-88], whether our findings can be generalized to the male population and whether there exits chronological interaction warrant further studies to clarify. Although the low frequency of *5-HTTVNTR *10-repeat allele in our sample (Han Chinese population) has been observed in other independent research groups [89,90], we acknowledged that false positive findings could occur due to skewed allele distribution. Replication of our results in a larger sample size or in other races with more balanced *5-HTTVNTR *10-repeat distribution is encouraged.

## Conclusion

The serotonin transporter plays a key role in regulating the synaptic serotonin level. The serotonergic system also exerts a broad influence on the stage of neurodevelopment. This is the first study using EEG to investigate the effect of *5-HTTLPR *and *5-HTTVNTR *polymorphism on local brain dynamics in the resting state. Our analyses revealed a global trend of reduced regional power, regardless of spectral bands, for carriers of the *5-HTTVNTR *10-repeat allele and those with the *5-HTTLPR *S-allele homozygotes. We speculated that the *5-HTTLPR *S-allele and *5-HTTVNTR *10-repeat allele contributed to less synchronized neural circuits at the local cortical region. This proposition was endorsed by previous structural imaging studies that revealed detrimental effects associated with the 5-HTTLPR S-allele on the neural architecture. The implications of our finding might be relevant to understanding the neural mechanism underlying 5-HTT genetic vulnerability across a broad range of psychiatric disorders.

## Competing interests

The authors declare that they have no competing interests.

## Authors' contributions

All the authors contributed to the conception and design of this project. Author TJ Chen initiated and designed the study and determined the protocol. Author TW Lee managed the literature searches and statistical analyses, and wrote the first draft of the manuscript. Authors YWY Yu and HC Wu executed the experiment protocol and undertook the EEG data collecting procedure, subject evaluation and quality control. Authors CJ Hong and SJ Tsai were responsible for the genetic material collection and analysis. All authors contributed to and have approved the final manuscript.
